# Two years retrospective study of maxillofacial trauma at a tertiary center in North West Ethiopia

**DOI:** 10.1186/s13104-017-2670-1

**Published:** 2017-08-08

**Authors:** Amare Teshome, Getaneh Andualem, Rediet Tsegie, Samuel Seifu

**Affiliations:** 1 Department of Dentistry, College of Medicine and Health Sciences, University of Gondar, Gondar, Ethiopia; 2 Department of Stomatology, College of Medicine and Health Sciences, Mekelle University, Mekelle, Ethiopia

**Keywords:** Maxillofacial trauma, Etiology, Treatment outcome, Interpersonal violence, Maxillofacial fracture

## Abstract

**Background:**

Maxillofacial injury poses a challenge to oral and maxillofacial surgeons working in developing countries with limited resource and human power. The present study aimed to determine the etiology, pattern, and management of maxillofacial trauma in Gondar university of Gondar hospital.

**Methods:**

A retrospective descriptive study design was used. Medical registration retrieving of patients with maxillofacial trauma visited dental center of University of Gondar Hospital from September 2013 to August 2015 was done. During data collection, etiology of trauma, pattern of fracture, treatment modality and complications were recorded using predesigned data collection template and analyzed using SPSS computer software version 20. Statistical analysis was done to show the sex distribution of maxillofacial trauma and the effect of alcohol intake on the incidence of trauma.

**Results:**

During 2-year period, September 2013–August 2015, 326 patients of maxillofacial trauma were treated in the dental center of university of Gondar hospital. The mean age was 29.12 (± 8.62) with age range of 11–75 years. Majority of the study participants (47.2%) were within the age group of 21–30 years. Eighty percent of the participants were male with a male to female ratio of 4.02:1. Interpersonal violence (75.8%) and Road traffic accident (21.5%) were the leading causes. Males are at high risk of maxillofacial trauma relative to females (P < 0.0001). There was high incidence of trauma in the weekend, rural residents, December to February, mandibular fractures and soft tissue injuries were the most common injuries. There was an associated injury in 79 (24.2%) patients in head and neck area, thoracic, abdominal and extremities. Half of the patients were managed conservatively (49.7%) with debridement and suture, while 45.7% of the patients were closed reduction and 4.6% were surgical open reduction. There were 25 post procedure complications especially in mandibular fractures.

**Conclusion:**

Interpersonal violence was the major cause of maxillofacial trauma, while mandible and soft tissue were the most affected maxillofacial areas. The federal ministry of health, Ethiopia should have well-organized maxillofacial center in tertiary hospitals for emergency management to avoid morbidity and mortality.

## Background

An injury is defined as “a physical injury that occurred when human body sustained sudden force in amount that is beyond the verge of physical tolerance “or the result of a lack of one or more vital elements, such as oxygen” [1].

The presence of trauma in the maxillofacial (MF) area have a substantial impact in the psychology and aesthetic of the patient and lays an everlasting effect on the behavior of the patient [2]. The presence of morbidity and mortality due to maxillofacial trauma is high even if there was a good recovery [3].

There is a varied etiology of maxillofacial trauma that differs from country to country due to cultural, socioeconomic and environmental factors [4, 5]. Road traffic accidents, violence, fall and sport activities were the leading causes of maxillofacial trauma [6–11]. Interpersonal violence is the leading cause of maxillofacial trauma in developed countries, while road traffic accident is the leading in developing countries [7].

The incidence of maxillofacial trauma was high in males with male to female ratio of 5:1 in Zimbabwe [8] and in the age of 19–28 [10]. A study done in Braille showed that there was 90.9% of maxillofacial fracture in patients sustained maxillofacial trauma and maxillary bone fracture was the most commonly occurred fracture [10]. however, other study in Iran showed high incidence of mandibular fracture with a common site of body of the mandible [11].

Maxillofacial trauma involves soft tissues of the face and bones of the mandible, mid-facial and frontal bones [12]. the severity and pattern of the MF trauma depends on the anatomic site of trauma, magnitude of the force and direction of the force [4, 12].

The maxillofacial treatment depends on the pattern and severity of the trauma and may be conservatively with debridement and suture, closed reduction with arch bar or eyelets, or surgically with open reduction. Procedures with open reduction resulted satisfactory facial aesthetic, shortened duration of work absence, and preserves function early and reduced the incidence of complications [13].

Comprehensive data on the etiology and pattern of maxillofacial injuries is not readily available in Ethiopia. The aim of this study was to determine the etiology, pattern of trauma and management modalities on traumatic patients visited university of Gondar dental center.

## Methods

We conducted a retrospective study in patients with maxillofacial trauma who treated in the dental center of university of Gondar hospital from September 2013 to August 2015. The hospital was founded in 1930s and serves as a tertiary hospital for the last 80 years. It is considered as a center of excellence teaching center in the northwest of Ethiopia. Patients with full-recorded medical record and above 10 years were included in the study. During the 2 years, three hundred seventy eight patients with maxillofacial trauma were treated in the dental center. However, fifty-two cases had incomplete record and excluded from the study. Data were collected from clinical notes from September 2015 to January 2016 using predesigned templates by three dental surgeons under the supervision of the principal investigator.

The data collected from the patient’s record were; age, gender, cause of trauma, anatomic site of fracture, associated injuries, and type of treatment modality. The etiology of trauma was divided into four main categories: (i) violence, which include interpersonal violence and weapons; (ii) road traffic accidents involving automobiles, motorcycles and pedestrians; (iii) sport injuries; and (iv) fall; fall from ground or high level.

Maxillofacial fractures were diagnosed with clinical and imaging examination (conventional radiograph or CT scan). Maxillofacial injuries were classified according to location of trauma, types of injury, and etiology.

This research is in accordance with all ethical standards, and the ethical review committee of university of Gondar approved the project. Permission to review the medical records of the patients was also provided by the director of Gondar university hospital.

The collected data were coded and entered into Epi info version 7. Then it exported to IBM SPSS 20 (IBM Corp, Armonk, NY, USA) for analysis. Descriptive statistics were used to summarize the sociodemographic characteristics, cause of trauma, pattern of trauma/fracture and the type of complications.

## Results

### Participant characteristics

Three hundred twenty six patients with maxillofacial trauma were seen at dental clinic of university of Gondar between September 2013 and August 2015. Overall, 261() patients were male with a male to female ratio of 4.02:1. The mean age of the participants was 29.12 (±8.62) years (range 11–75 years) and the most commonly affected age group was 21–30 years (47.24%). The incidence of trauma was high in rural residents (Table 1).

**Table 1:** Frequency of the etiology of maxillofacial injuries in relation to age group and sex (n = 326) among patients seen in dental center of university of Gondar hospital, 2013–2015

	Etiology			P value/Chi square
	Interpersonal violence	Road traffic accident	Others (fall, sport etc.)	
Gender				
Male	211	44	6	P < 0.0001
Female	36	26	3	X^2^ = 18.44
Age				
11–20	50	16	3	P < 0.00001 x^2^ = 66.0355
21–30	117	32	5	
31–40	40	13	1	
>40	9	39	1	
Residency				
Urban	57	48	4	P < 0.00001
Rural	190	22	5	x^2^ = 51.2267

### Cause of maxillofacial trauma

The major cause of maxillofacial trauma were; interpersonal violence (IPV) (75.8%; n = 247), road trafficking accident (RTA) (21.5%, n = 70) and others (2.7%, n = 9). Interpersonal violence was the leading cause of maxillofacial trauma in patients who take alcohol. Moreover, young patients (11–30 years) were mainly affected by IPV whereas middle and old age (>40 years) were of RTA (Table 1). Almost all (87.6%) cases of maxillofacial trauma incidents in the rural residents was due to IPV (Table 1).

### Alcohol intake and maxillofacial trauma

Overall, alcohol intake was recorded in 35.6% of the patients, mainly in males (92.2%) (P < 0.0001). Males and 21–30 years old patients were taken alcohol such as; beer, wine, whisk, traditional drinks (Tela, Areki, Teji) etc. before the incidence of the trauma (Table 2).

**Table 2:** The association between alcohol intake and maxillofacial trauma incidence among patients visited dental center of university of Gondar Hospital, 2013–2015

Sociodemographic characteristics	Maxillofacial trauma		P value/Chi square
	Alcohol taken	No alcohol	
Gender			
Male	107 (92.2%)	1549 (73.3%)	P < 0.0001 X^2^ = 16.7353
Female	9 (7.8%)	56 (26.7%)	
Age			
11–20	27 (23.3%)	42 (20%)	P < 0.001 X^2^ = 18.7621
21–30	67 (57.6%)	87 (41.5%)	
31–40	17 (14.7%)	37 (17.6%)	
>40	5 (4.4%)	44 (20.9%)	

### Time lag between trauma incidence and dental visit

**Fig. 1:**
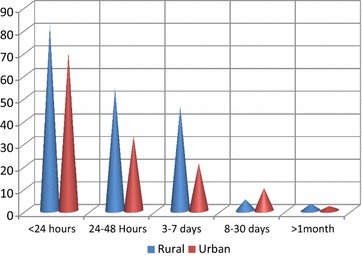
Frequency of time lag between trauma incidences to dental visit according to residency (n = 326) among patients visited dental clinic of Gondar university, 2013–2015

**Fig. 2:**
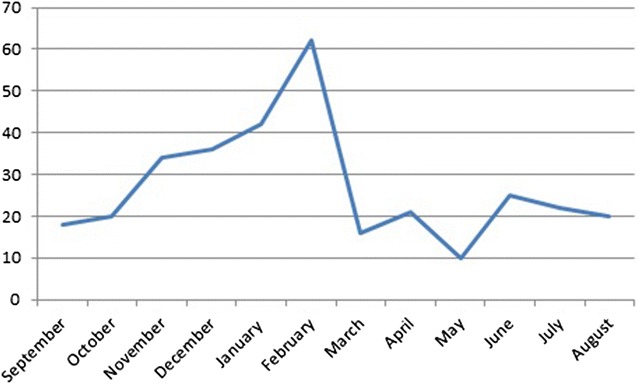
Monthly distribution of maxillofacial trauma incidence among patients visited dental center of university of Gondar hospital, 2013–2015

The majority of patients (46.6%) arrived to dental clinic within 24 h. Daytime injuries occurred in majority of the cases (71.62%) (Fig. 1). Most of the cases were occurred in the weekend (39%), and from December to March with a peak level on February (Fig. 2).

### Pattern of maxillofacial trauma

Of the 326 maxillofacial injuries, 162 (49.4%) were only soft tissue injuries; laceration, contusion, and abrasion. Maxillofacial fracture occurred in 164 (50.61%) patients, of these, 123 (75%) of patients had mandibular fracture (Table 3).

**Table 3:** Pattern of maxillofacial fracture among patients visited dental clinic of university of Gondar hospital (n = 164), 2013–2015

Type of fracture	Frequency	Percent (%)
Mandibular fractures	*123*	*75*
Condyle	16	9.8
Ramus	10	6.1
Angle	19	11.6
Body	54	32.9
Symphysis/parasymphysis	24	14.6
Nasal fractures	3	1.8
Frontal fractures	1	0.6
Zygomatic fractures	4	2.4
Maxillary fractures	*33*	*20.2*
Le fort I	27	16.5
Le fort II	5	3.1
Le fort III	1	0.6

### Associated injuries

The concomitant injuries were observed in 79 (24.23%) of patients with maxillofacial injury. Of these, extremities (43.04%; 52.3% were in the upper extremities) and head and neck (31.65%) were commonly affected (Table 4).

**Table 4:** Associated injuries among patients visited dental clinic of university of Gondar hospital (n = 79), 2013–2015

Associated injuries	Frequency	Percentage (%)
Head and neck injury	25	31.7
Thoracic injury	8	10.1
Abdominal injuries	12	15.2
Extremities injuries		
Upper extremities	23	29.1
Lower extremities	11	13.9
Total	79	100

### Treatment modalities

Two hundred sixty-three (80.67%) patients were required surgical treatment, of which debridement (76.53%) was the most common surgical procedure performed. Out of 164 patients with maxillofacial fractures, 149 (90.85%) patients treated by closed reduction using mandibulo–maxillary fixation, either with eyelet wiring methods or arch bars while 15 (9.15%) patients were managed by open reduction and intraosseous wiring. The patients were reviewed for 6 months (every 2 week for the first 6 weeks and every month from 6 week to 6 months).

### Post treatment complications

In the 2 years maxillofacial trauma cases, complications occurred in 25 patients (7.7%). Most of the complications were in the mandible: at the angle (7 cases), symphysis (3 cases), body (6 cases), and body and angle (3 cases) while six cases were occurred in the maxilla. The complications were; malocclusion, surgical site infection, chronic sinusitis and non-union. Malocclusion (48%) and trauma site infections (28%) were the most common complications recorded (Table 5).

**Table 5:** Type of post treatment complications among maxillofacial trauma patients visited university of Gondar hospital (n = 25), 2013–2015

Complications	Frequency	Percent (%)
Malocclusion	12	48
Surgical site infection	7	28
Chronic sinusitis	2	8
Non-union	4	16

## Discussion

Trauma, the leading cause of death in the first 40 years of lie, could be considered as the cause of productivity loss due to loss of working hours than cancer and heart diseases combined. Maxillofacial traumas are a common part of the multiple traumas resulting from road traffic accident, assault, sport, and fall. This cause differs from country to country due to cultural, environmental and socioeconomic factors [6, 14].

The incidence of maxillofacial trauma was fairly constant with seasonal variation as showed in the literature [15, 16]. However, in the present study the incidence was high from October to March with a peak level on February which coincides with a study done in Iran [11] and western Nepal [17]. This might be due to the existence of many public holidays in the country during this months and majority of the community spend their time work free and takes alcohol as recreation. Moreover, there was high incidence of trauma in the weekend (Saturday and Sunday).

The morbidity and mortality of traumatic patients is depend on their arrival time to the health institution. Majority (46.6%) of the patients visited university of Gondar dental clinic with in 24 h of trauma exposure. It agrees with a study conducted in Nigeria [18] and France [19].

The sex distribution of maxillofacial trauma incidence is highly frequent in males and is coincides with studies conducted elsewhere [17, 19–24]. The overall male to female ratio was 4.02:1 which corresponds with the study done in India (4.2:1) [25], and Zimbabwe (5:1) [8]. This might be due to that men tend to be more often involved in aggressive and conflict-ridden situations and are mostly involved in outdoor activities than women.

This study showed high incidence of trauma in young adults in their third decades (21–30 years) which is in agreement with many other studies [9, 11, 20, 23–27]. This may reflect this age group is more energetic and aggressive than others. More over, rural residents were more affected by maxillofacial trauma than urban residents. This is due to the existence of revenge, rural residents spent there time free from work from December to March and engaged in alcoholic drinks.

The present study revealed that interpersonal violence (75.8%) was the leading cause of maxillofacial trauma. In this respect it agrees with studies conducted in Kenya [27], Brazil [4], France [19], Bulgaria [28], and Johannesburg [29]. However, some other literature [7, 9, 11, 18, 22, 23, 30, 31] showed RTA as a major cause of maxillofacial trauma. This result may reflect the fact that majority of the participants were rural residents and adults.

This study revealed 1/3rd of the patients had alcoholic ingestion before the incidence of maxillofacial trauma. This result supported the hypothesis that alcohol intake and maxillofacial injury in adults has a strong relationship [14, 32]. There was a significant association between alcohol intake and maxillofacial trauma in males (P < 0.0001) and adults (21–30 years) (P = 0.0003). This is because of the impact of alcohol on balance, and brings aggression.

When maxillofacial area is injured, mandible is the most vulnerable bone than the mid facial bones [11, 18, 19, 24, 25, 30]. The present study revealed that 75% of maxillofacial bone fractures was occurred in the mandible. This fractures was common in body (32.9%), symphysis/parasymphysis (14.6%) and angle (11.6%) of the mandible. This could be due to the mandible is mobile and has less bony support.

This study revealed high incidence of concomitant injuries to the head and neck (31.7%) and, upper limb (29.1%). This finding is inline with previous studies that showed high incidence of injury to the limb [33, 34]. This is due to the victims use their arm to defend themselves from assaults.

Previous studies done in Iran [11] and South Africa [29] showed that majority of maxillofacial fractures were treated with open reduction and has low post operation complications. The present study is not inline with this findings where only 9.15% of the patients had open reduction. This variation is because of the absence of qualified experts in maxillofacial surgery in the study area. However, a study done in Nigeria has similar finding with our studies [18]. Surgical management of maxillofacial fracture with open reduction promises a shortened period of bone fixation, rapid restoration of anatomy and function, bony union with less callus formation [35].

There was 15.2% of complications observed after 6 months followup period which is lower than the result found by Chalya et al. [36]. The presence of this much incidence of complication may be due to most procedures are done by closed reduction due to the absence of maxillofacial surgeons.

In Ethiopia, the service of maxillofacial and dentistry in general is neglected due to the absence of qualified maxillofacial surgeons, governments priority to infectious diseased and the absence of dental and maxillofacial equipment in the country. The absence of experts in oral and maxillofacial and plastic surgery for internal fixation and open reduction are a major obstacles in achieving early healing and restoration of cosmetics in facial trauma patients.

Limitation of this study was; some of patient medical records were incomplete and were excluded from the study. Therefore a larger sample size prospective study should be done to make a conclusive finding on the epidemiology of maxillofacial trauma in Ethiopia.

## Conclusion

Interpersonal violence and road trafficking accidents were the leading causes of maxillofacial trauma. Males and 21–30 years aged were the most affected groups, while mandibular fracture and soft tissue injuries were the commonly occurring type of injuries. Majority of maxillofacial fractures were managed by closed reduction.

## Abbreviations

IPV: interpersonal violence
RTA: road traffic accident
MF: maxillofacial
